# Nitrogen doped graphene with diamond-like bonds achieves unprecedented energy density at high power in a symmetric sustainable supercapacitor†

**DOI:** 10.1039/d1ee02234b

**Published:** 2022-01-07

**Authors:** Veronika Šedajová, Aristides Bakandritsos, Piotr Błoński, Miroslav Medveď, Rostislav Langer, Dagmar Zaoralová, Juri Ugolotti, Jana Dzíbelová, Petr Jakubec, Vojtěch Kupka, Michal Otyepka

**Affiliations:** Regional Centre of Advanced Technologies and Materials, Czech Advanced Technology and Research Institute (CATRIN), Palacký University Šlechtitelů 27, 783 71 Olomouc Czech Republic michal.otyepka@upol.cz a.bakandritsos@upol.cz; Department of Physical Chemistry, Faculty of Science, Palacký University 17. listopadu 1192/12 779 00 Olomouc Czech Republic; Nanotechnology Centre, Centre of Energy and Environmental Technologies, VŠB–Technical University of Ostrava 17. listopadu 2172/15 Poruba 708 00 Ostrava Czech Republic; Department of Experimental Physics, Faculty of Science, Palacký University Olomouc 17. listopadu 1192/12 Olomouc 77900 Czech Republic; IT4Innovations, VŠB–Technical University of Ostrava 17. listopadu 2172/15 708 00 Ostrava-Poruba Czech Republic

## Abstract

Supercapacitors have attracted great interest because of their fast, reversible operation and sustainability. However, their energy densities remain lower than those of batteries. In the last decade, supercapacitors with an energy content of ∼110 W h L^−1^ at a power of ∼1 kW L^−1^ were developed by leveraging the open framework structure of graphene-related architectures. Here, we report that the reaction of fluorographene with azide anions enables the preparation of a material combining graphene-type sp^2^ layers with tetrahedral carbon–carbon bonds and nitrogen (pyridinic and pyrrolic) superdoping (16%). Theoretical investigations showed that the C–C bonds develop between carbon-centered radicals, which emerge in the vicinity of the nitrogen dopants. This material, with diamond-like bonds and an ultra-high mass density of 2.8 g cm^−3^, is an excellent host for the ions, delivering unprecedented energy densities of 200 W h L^−1^ at a power of 2.6 kW L^−1^ and 143 W h L^−1^ at 52 kW L^−1^. These findings open a route to materials whose properties may enable a transformative improvement in the performance of supercapacitor components.

## Abstract 2

A nitrogen-superdoped graphene with diamond-like interlayer bonds is obtained *via* the radical chemistry of fluorographene, affording a symmetric supercapacitor with an energy density of 200 W h L^−1^ at a power of 2.6 kW L^−1^ and 143 W h L^−1^ at 52 kW L^−1^.

## Graphical abstract

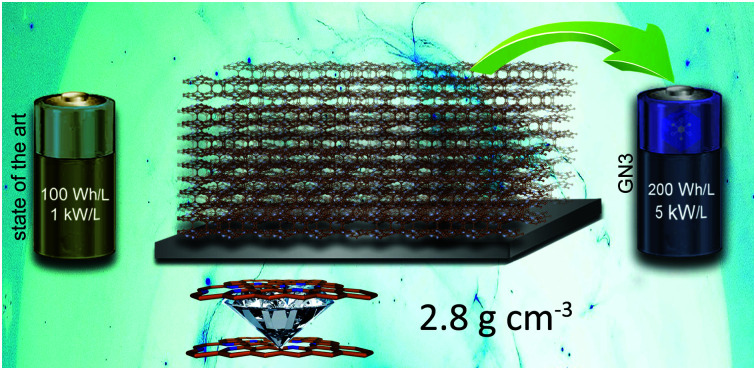

**Broader context**

Modern society relies on electricity. The demand is bound to grow due to the increasing electromobility, the number of mobile devices, and extending the networks for the internet of things. The depleting reserves of fossil-based energy resulted in efforts to support renewable resources, which are, however, intermittent in their production. These facts call for the development of electrochemical energy storage devices with improved performance, safety, eco-friendliness, and lower cost in order to contribute to the goal of the United Nations for affordable, reliable, and sustainable energy. Lithium-ion batteries have matured and currently dominate the field. Nevertheless, carbon-based supercapacitors offer independence of critical elements, alongside safety, long life-cycle, and ultrafast charging–discharging. Here, we present a nitrogen superdoped graphene material with diamond-like interlayer bonds that dramatically increases the energy content, which can be stored per volumetric unit of the electrode—the Achilles heel of contemporary supercapacitors. The electrode displays an ultrahigh mass density compared to porous carbons, keeping intact its ability to host the electrolyte ions—the energy carriers. Consequently, a supercapacitor device made from this electrode delivers energy density twice as high as that of top-rated materials and several-fold higher than commercial supercapacitor carbons, thus enhancing the performance of supercapacitor components.

## 1. Introduction

Supercapacitors are energy storage devices with remarkable qualities including fast charging/discharging (*i.e.* high power) and extralong cycle-life.^1^ Unfortunately, the energy density of the best existing supercapacitors (*i.e.* their ability to store charge/energy) is low. Commercial supercapacitors have cell-level specific energies (and energy densities) of 10 W h kg^−1^ (5–8 W h L^−1^),^2,3^ while lead–acid batteries offer 20–35 W h kg^−1^ (40–80 W h L^−1^)^4^ and state-of-the-art Li-ion batteries achieve ∼150 W h kg^−1^ (∼250 W h L^−1^).^5,6^ However, Li-ion batteries suffer from long charging times and, unlike supercapacitors, undergo irreversible processes during cycling that gradually reduce their energy density and thus their cycle-life. To exploit the benefits of supercapacitors in a broader range of applications, it will be necessary to identify electrode materials that have substantially improved energy densities combined with long life and high power. In addition, replacing metal atoms in electrode materials with non-metal and earth-abundant elements, such as carbon, would have significant environmental advantages, reducing our reliance on critical natural resources and increasing sustainability.

Due to the importance of the electrode material/electrolyte interface for charge storage,^7^ intense efforts have been focused on lightweight materials with high surface areas such as nitrogen-doped mesoporous carbon^8^ (2000 m^2^ g^−1^), carbon nanosheets^9^ (2500 m^2^ g^−1^), activated graphene^10^ (3100 m^2^ g^−1^), and carbon nanotubes^11^ (1300 m^2^ g^−1^). The specific energies of these materials range from *ca.* 10 to 90 W h kg^−1^, with the highest values being reported for activated graphene,^10^ carbon nanosheets,^9^ and nanotubes^11^ in ionic liquid (IL)-based electrolytes. Unfortunately, like most commercial electrodes,^12^ these carbon materials have very low mass densities (*ca.* 0.3–0.7 g cm^−3^).^8,10^ Consequently, the energy densities achieved with mesoporous carbon,^8^ activated graphene,^10^ and carbon nanosheets^9^ (or single wall carbon nanotubes^11^) are only 22, 26, and 45 W h L^−1^, respectively.

To achieve higher energy densities, which is a key performance parameter,^2–4,13–16^ efforts have been made to increase the mass density of electrode materials. Compressing a graphene electrode increased its mass density from 0.34 to 0.75 g cm^−3^ and its energy density from 26^10^ to 48 W h L^−1^ (ref. 17) without adversely affecting the interactions between the electrolyte ions and the carbon surface. Capillary densification of a chemically reduced graphene gel in the presence of an IL led to an even higher density of 1.3 g cm^−3^, resulting in a material that delivered 90 W h L^−1^ at a power density of 1.1 kW L^−1^ (1 A g^−1^).^18^ It was deduced that densification in the presence of the non-evaporating IL prevented the restacking of the graphene sheets and helped preserve the material's charge transport properties. Mechanical compression of a H_2_O_2_-treated reduced graphene oxide^19^ yielded a material with a density of 0.7 g cm^−3^ and a holey structure (beneficial for ion diffusion) that very effectively promoted three-dimensional ionic transport, delivering 85 W h L^−1^ at 1.75 kW L^−1^ (1 A g^−1^). In 2016, capillary drying was combined with a different pore-forming agent (ZnCl_2_), to afford a monolithic dense (0.9 g cm^−3^) graphene electrode^14^ exhibiting 60 W h L^−1^ at 0.4 kW L^−1^ (0.6 A g^−1^). Further attempts to increase the energy density by heteroatom tri-doping^20^ and densification^21^ were not more effective, resulting in energy densities of 40 and 65 W h L^−1^, respectively. Even high mass density inorganic phases such as 1T-MoS_2_ did not exceed 80 W h L^−1^ at 1.12 kW L^−1^ (0.5 A g^−1^).^22^ The highest energy density reported to date was obtained using electrodes consisting of interdigitated bilayers of exfoliated graphene-mediated hydrogen iodide-reduced graphene oxide (EGM-GO)^23^ with a mass density of *ca.* 1 g cm^−3^ and a capacitance of 203 F cm^−3^. These electrodes offered an energy density of 113 W h L^−1^ at 0.9 kW L^−1^ (1 A g^−1^) (see the experimental section for information on the metrics used). Thus, over the last decade there have been small improvements in materials design for higher energy contents, and power densities have remained relatively low.

Here we report a carbon-based electrode material, GN3, with an unprecedented density of 2.8 g cm^−3^ and an N_2_ sorption-based surface area of 128 m^2^ g^−1^ that can host ions even more efficiently than carbon materials with surface areas exceeding 2000 m^2^ g^−1^. GN3, which is prepared by reacting graphite fluoride with sodium azide, has tetrahedral (sp^3^) C–C bonds, which were identified by solid-state nuclear magnetic resonance in the same region as the C–C bonds in diamond. However, it retains a 2-D structure with a very high content of aromatic (and thus conductive) regions, together with nitrogen superdoping in the vacancies and holes of the aromatic lattice. The ultrahigh mass density of GN3 combined with its polar nitrogen moieties and vacancies facilitated an energy density of 200 W h L^−1^ at a power density of 2.6 kW L^−1^, corresponding to improvements of 74% and 190%, respectively, over the previous record.^23^

## 2. Results and discussion

Motivated by the importance of fluorine and radical chemistry^24,25^ in the synthesis of sp^3^-rich carbon materials,^26–28^ and by the high density of such materials,^29^ we hypothesized that fluorographene chemistry could produce carbon derivatives with high mass densities. This hypothesis was strengthened by the fact that in (C_2_F)_*n*_, whereby fluorine atoms occupy one side of every other carbon sheet in an FCCF manner, the carbon atoms in between adopt a diamond-like structure,^30,31^ ascribing high mass density.^31^ The formation of similar sp^3^-rich structures was also verified theoretically and experimentally for bilayer graphene.^32,33^ Despite their high mass density, fluorocarbons are large band-gap insulators^34^ and lack sites capable of interacting strongly with ionic species and facilitating their transport. However, because the defluorination and functionalization of fluorographene is known to occur *via* radical reactions propagated by fluorine elimination,^35,36^ these processes could potentially be exploited to drive sp^3^ C–C bond formation and create graphene-based materials with high mass density.

**Fig. 1:**
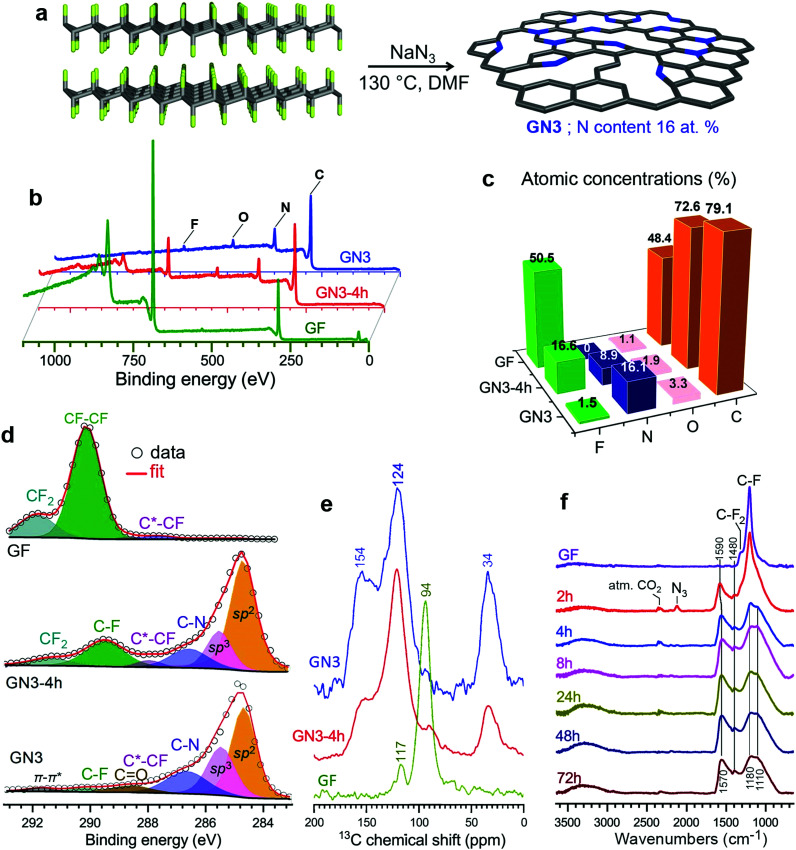
Synthesis and characterization of the GN3 material. (a) Schematic depiction of the synthesis of GN3 from sonicated graphite fluoride (GF). (b) XPS survey spectra of the starting GF and the N-doped derivative after reaction times of 4 h and 72 h (GN3). (c) Elemental compositions of the same materials determined by XPS. (d) Deconvoluted HR-XPS spectra for the C 1s regions. (e) CP MAS ^13^C solid state NMR spectra and (f) IR spectra of GF, reaction intermediates at various time points, and the final GN3 product.

To investigate this hypothesis, we experimentally and theoretically probed the reaction of few-layered fluorographene with sodium azide as a defluorinating agent that could at the same time introduce nitrogen atoms into the formed structure. This would increase the polarity of the carbon surface and create vacancies, as previously observed following reactions of fluorographene with various nitrogen-containing nucleophiles.^36–38^ Sonicated bulk graphite fluoride reacted very efficiently with NaN_3_ in dimethylformamide at 130 °C, resulting in nitrogen superdoping (Fig. 1a). X-ray photoelectron spectroscopy (XPS) revealed a decrease in the material's content of F atoms after a reaction time of 4 h and almost complete elimination of F after 72 h, at which point the material's N content reached 16.1 at% (Fig. 1b and c). This change was reflected in the C 1s regions of the materials’ XPS spectra (Fig. 1c): the initially F-bonded carbon atoms of fluorographene, with binding energies above 289.5 eV, were transformed into (i) aromatic sp^2^ carbons (284.7 eV, 45%), (ii) non-functionalized sp^3^ carbons (285.5 eV, 25%), and (iii) nitrogen bonded carbons (286.6 eV, 19%). The other components in this spectral region were attributed to small amounts of residual fluorine and oxygen from the environment. The HR-XPS spectra of the N 1s envelope (Fig. S1, ESI†) revealed the presence of nitrogen atoms in pyridinic and pyrrolic configurations (or protonated and non-protonated N centres), as well as a very small number of graphitic nitrogens (44, 49, and 7 at%, respectively). The dominance of pyridinic and pyrrolic nitrogens is consistent with the vacancies present in the parent material^36^ and with the extensive development of such vacancies during defluorination.^36–38^

**Fig. 2:**
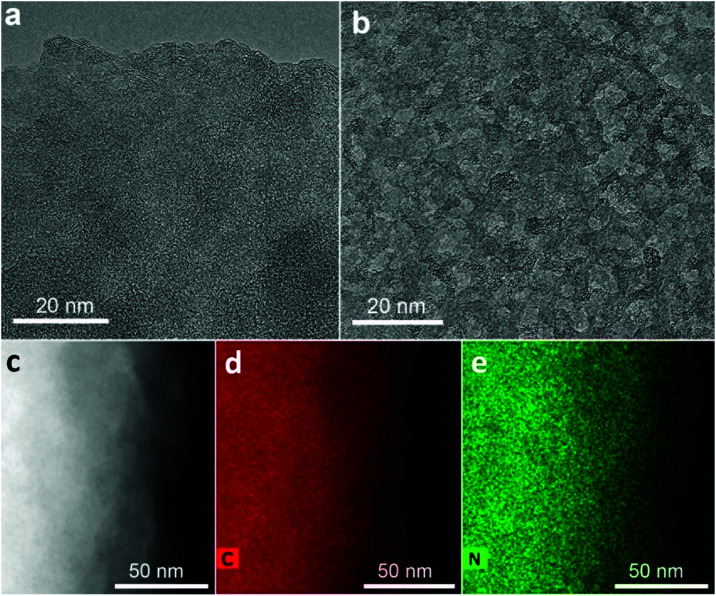
(a) and (b) High-resolution transmission electron microscopy images of GN3 flakes; several areas had extensively holey structure, as shown in (b), and in Fig. S1b (ESI†). (c) HAADF image of a GN3 flake used for EDXS mapping, along with the corresponding (d) carbon and (e) nitrogen map.

The ^1^H → ^13^C CP MAS solid-state NMR spectra of the GN3-4 h intermediate and GN3 show peaks at 34 ppm (Fig. 1e). Chemical shifts in this range are typical for non-functionalized and non-nitrogen bonded sp^3^ carbons in diamond and diamond-like carbon materials.^39^ This peak was stronger in the spectrum of GN3 than the GN3-4 h intermediate, indicating that these sp^3^ carbons formed gradually as the reaction progressed. Furthermore, the peaks centred at 124 and 154 ppm indicate the presence of a π-conjugated aromatic network and aromatic >C=N moieties (pyridinic and pyrrolic),^39,40^ respectively. ^19^F → ^13^C CP MAS of the starting GF revealed peaks corresponding to CF_2_ (117 ppm) and CF (94 ppm) groups, typical for FG.^41^ Such non-functionalized tetrahedral carbons at 34 ppm are not detected in graphene oxide, reduced graphene oxide, or graphene.^42,43^ The reaction's progress was also verified by infra-red spectroscopy (Fig. 1f). Specifically, the bands of the CF and CF_2_ groups of GF (1200 and 1305 cm^−1^, respectively) were progressively replaced with bands at 1580 and 1210 cm^−1^ (characteristic of aromatic carbon rings^44^), indicating the formation of an sp^2^ network. Additional aromatic-ring vibrations, appearing at 1400 cm^−1^, could be ascribed to heteroatom substitution (*e.g.* with pyridinic nitrogens^44,45^), as suggested by theoretical calculations.^46,47^ The Raman spectrum of GN3 featured broad D and G bands at 1300 and 1590 cm^−1^, respectively, and an *I*_D_/*I*_G_ ratio of 1.3, which remained unchanged even after heating at 1000 °C in an argon atmosphere (Fig. S2, ESI†), indicating the presence of a large number of non heat-susceptible sp^3^ carbons and non-healable defects (*i.e.* vacancies). Raman bands and X-ray diffraction peaks (XRD) deconvolution performed on GN3, and on the commercial porous carbon for comparison (Fig. S3 and S4, ESI†), showed that the GN3 displays a disordered structure with randomly developed tetrahedral C–C bonds. Results also highlighted the very small planarity of the aromatic areas, with a lateral size (*L*_a_) of *ca.* 4 nm (Fig. S3 and S4 (ESI†), and comments in the caption). Further insights into the N-doping of fluorographene with NaN_3_ were obtained through density functional theory (DFT) calculations. The N_3_^−^ anion initiated the reaction by nucleophilic attack on carbon radical defects, leading to N_2_ release and fluorine elimination (Fig. S5, ESI†). The attachment of azide groups in the initial stages of the reaction was confirmed by the infra-red spectrum of the 2 h intermediate (Fig. 1f). High-resolution transmission electron microscopy (HR-TEM, Fig. 2a and b) revealed that GN3 indeed exists as sheets with patches and holes.

Energy dispersive X-ray spectroscopy (EDXS) elemental mapping with high-angle annular dark-field scanning transmission electron microscopy (HAADF-STEM, Fig. 2c) showed that the GN3 sheets (Fig. 2d) are densely and homogeneously covered with nitrogen (Fig. 2e). Thermogravimetric and evolved gas analyses in air (Fig. S6, ESI†) indicated that these nitrogen atoms were embedded in the lattice rather than being out-of-plane functionalities, because emission of NO gas (*m*/*z* = 30) peaked at very high temperature (675 °C), at which CO_2_ emission also took place due to carbon lattice decomposition.

**Fig. 3:**
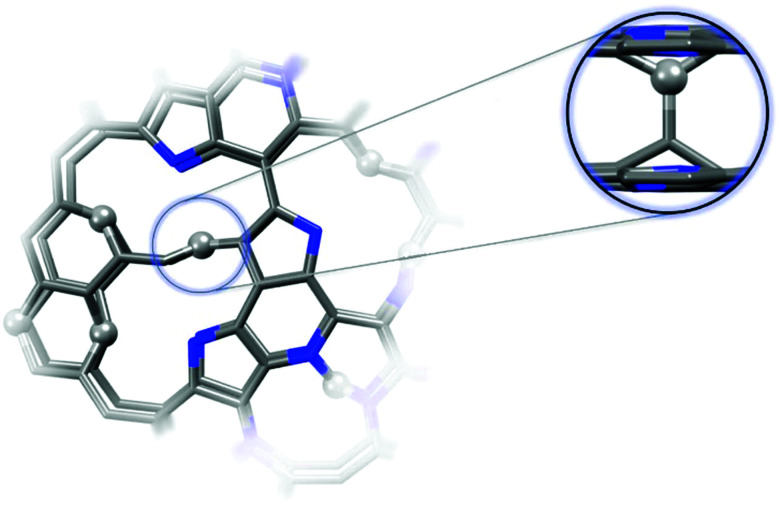
Theoretical model of GN3 structural fragment (C : N atomic ratio of *ca.* 84 : 16) optimized by first-principles spin-polarized DFT calculations. Top view of this structure with the carbons bearing radicals and forming interlayer bonds highlighted as spheres (zoomed side-view). The model simulates the structure locally (few-atom level) and does not (and cannot) provide macroscopic structural information.

To better understand the formation of the tetrahedral C–C bonds, theoretical models of GN3 sheet fragments were studied using spin-polarized DFT (Fig. 3), consistent with the experimental findings (*i.e.* containing vacancies and nitrogen dopants mainly in pyridinic and pyrrolic configurations, Fig. 3). Remarkably, the system relaxed into a thermodynamically stable structure with spontaneously formed tetrahedral sp^3^ C–C bonds, verifying the experimental NMR findings. The bonds were formed between the carbons in the pyridinic vacancies, where radicals were centred (highlighted by spheres in Fig. 3). Similar sp^3^ bonding was suggested to form after the introduction of atomic vacancies and pyrrolic N atoms by N-ion beam irradiation of graphene sheets, which creates carbon atoms with dangling bonds (radicals) around the vacancies.^48^

**Fig. 4:**
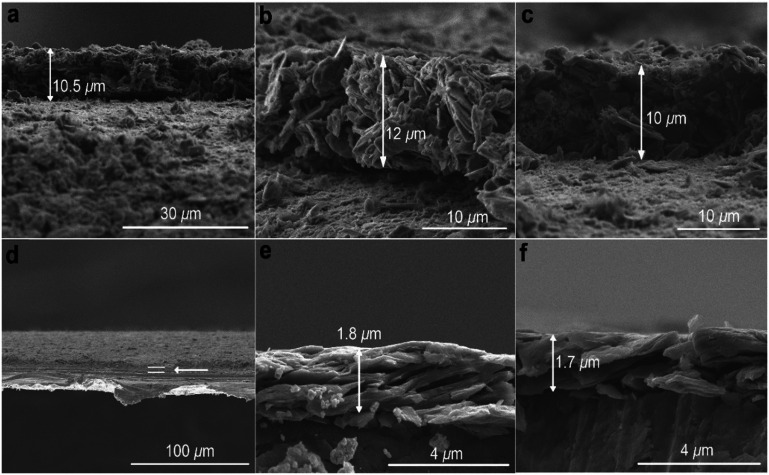
SEM images of GN3 with 10 mass% additives pasted on Al foil. (a)–(c) Before pressing and (d)–(f) after pressing. Pressing the Al foil itself did not affect its thickness (Fig. S7a and b, ESI†).

Films of GN3 or GN3 with additives (polymer binder 10%; conductive additive 5%) were formed by pasting slurries onto 15 μm-thick Al foils for density measurements and preparation of supercapacitor electrodes (ESI,† Methods). Scanning electron microscopy (SEM, Fig. 4a–c) showed that compression at 80 kN for 1 minute (∼65 MPa) reduced a *ca.* 10 μm thick film of GN3 to a thickness of 2 μm (Fig. 4d–f). From thickness measurements performed using SEM and a digital micrometer (Fig. S7c and d, ESI†), the density of these films consistently reached values of 2.7–2.8 g cm^−3^, compared to ∼0.5 g cm^−3^ before pressing. The same mass density was also attained for a high-mass loading electrode (8.3 mg cm^−2^, Fig. S7i and j, ESI†). The NMR spectrum of the pressed material was identical to that before pressing, indicating that pressing caused only bed consolidation, and not formation of bonds. Five GN3 batches from different reactions were measured to determine the mass density; it should be noted that densities were only measured after dialysis of GN3. Control tests were performed using the same procedure with Al foil alone (Fig. S7a and b, ESI†) and with commercial carbons of high surface area (Fig. S7e–h, ESI†), namely porous carbon (PC) from ACS Material (0.3 g cm^−3^, 2000 m^2^ g^−1^ according to N_2_ BET) and YP-80F Kuraray carbon (KC) (0.6 g cm^−3^, 2363 m^2^ g^−1^ according to N_2_ BET; also measured in-house, Fig. S8b and d, ESI†). The thickness measurements for the PC carbon were cross-checked by SEM (Fig. S9, ESI†). Results verified that no compression took place for Al foil, and that all mass density calculations for the commercial carbons after their pressing matched those given by the provider. Moreover, we performed elemental analysis for Na showing that 0.02 mass% of sodium remained in GN3, and therefore, the respective contribution in mass density of the material is negligible. The surface area of GN3 determined from the N_2_ sorption isotherm using BET equation was only 128 m^2^ g^−1^, (Fig. S8a and c, ESI†). The surface area determined by methylene blue sorption was 300 m^2^ g^−1^ (Fig. S10, ESI†), suggesting that under solvated conditions (as in an electrolytic supercapacitor cell), charged species/molecules, like methylene blue in this case, may penetrate into the structure of GN3. Interestingly, preliminary electrochemical testing of GN3 showed that pressing did not affect its charge storage properties; in fact, pressing increased the capacitance relative to the non-pressed electrode (Fig. S11, ESI†).

**Fig. 5:**
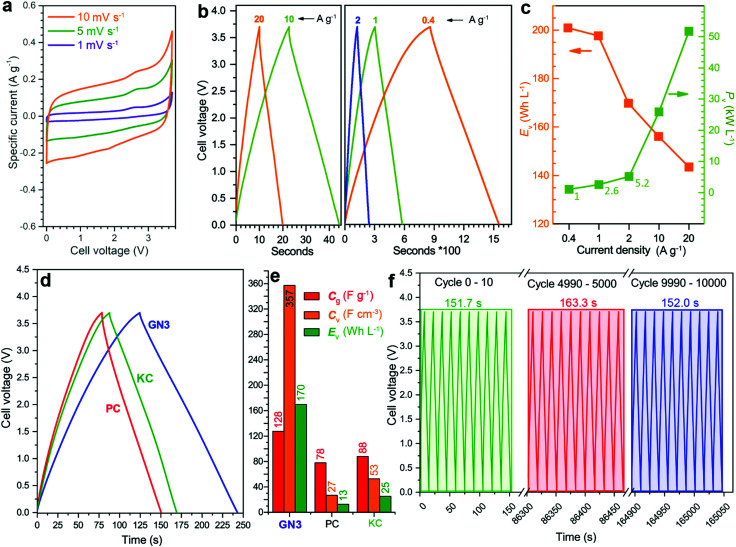
Electrochemical characterization of a symmetric supercapacitor cell with GN3 electrodes. (a) CV curves in the EMIM-BF_4_ and TTE (9 : 1) electrolyte at low scan rates. (b) GCD profiles at different specific currents. (c) Energy and power density of GN3 at increasing specific currents. (d) Comparison of the GN3 cell with symmetric cells made using commercial high surface area (2000 m^2^ g^−1^) porous carbons (PC and KC) at 2 A g^−1^ and (e) the performance of these cells. (f) Cyclic stability of GN3 showing the GCD profiles at the beginning, mid-point, and end of a 10 000 cycle test at 20 A g^−1^ current density.

The electrochemical properties of GN3 were studied in a symmetric full cell using as the electrolyte the IL 1-ethyl-3-methylimidazolium tetrafluoroborate (EMIM-BF_4_) with 1,1,2,2-tetrafluoroethyl-2,2,3,3-tetrafluoropropyl ether (TTE) in a 9 : 1 ratio. The cyclic voltammograms (CVs, Fig. 5a) were quasi-rectangular in shape with minor redox peaks, probably due to the nitrogen lattice atoms of the GN3 material.^49^ This shape was preserved even at very high scan rates (Fig. S12, ESI†), indicating that the system exhibits predominantly capacitive behaviour^10^ with fast charge transport in the material and at the interfaces.^15^ These features were verified by galvanostatic charge/discharge measurements (GCD, Fig. 5b), which yielded linear and symmetric profiles (124 s charging, 118 s discharging at 2 A g^−1^, 95% energy efficiency). The efficiency improved to 100% at 20 A g^−1^ (22 s charging, 22 s discharging). At 1 A g^−1^, the GN3 cell achieved an ultrahigh energy density of 197.6 W h L^−1^ at a power of 2.6 kW L^−1^. At 20 A g^−1^, the energy density remained high at 143.5 W h L^−1^, while the power density jumped to 51.8 kW L^−1^ (Fig. 5c).

**Fig. 6:**
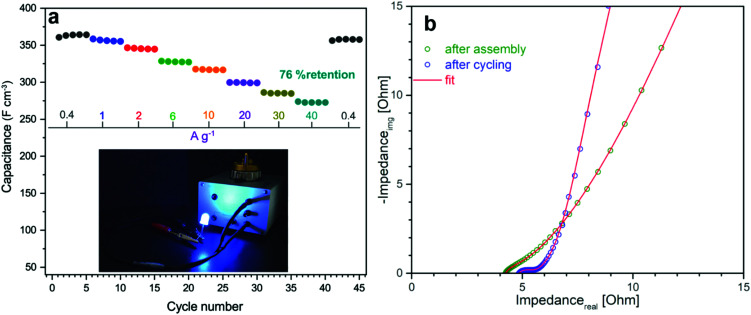
Electrochemical properties of a symmetric supercapacitor cell with GN3 electrodes. (a) Rate test at increasing specific currents. (b) Nyquist plots of GN3 after cell assembly and after cycling in EMIM-BF_4_ and TTE electrolyte (9 : 1 ratio).

For benchmarking, the carbons PC and KC (the latter widely used in commercial supercapacitors^50^) were evaluated under identical conditions. GN3 had a significantly better discharging time (Fig. 5d) than PC and KC, and its performance was superior in both volumetric and gravimetric terms (Fig. 5e), which is impressive given the dramatic differences in the materials’ BET surface areas. The cycling stability test of the GN3 material showed capacitance retention of 100% after 10 000 cycles at 20 A g^−1^ (Fig. 5f) and 98% after 14 000 cycles at 5 A g^−1^ (Fig. S15, ESI†). Rate testing of the GN3 cell showed that 76% retention of its capacitance at 40 A g^−1^ (Fig. 6a). A similar (70%) capacitance retention was achieved at 40 A g^−1^ for a cell made with exfoliated graphene-mediated HI-reduced graphene oxide (EGM-GO), which contained 50% exfoliated graphene.^23^

For comparative purposes, reported capacitance retention values for other high-performance graphene-based electrodes are *ca.* 65% at 40 A g^−1^ for liquid-mediated densified graphene,^18^ 70% at 40 A g^−1^ for holey graphene,^19^ 57% at 10 A g^−1^ for capillary-densified graphene,^21^ and 77% at 20 A g^−1^ for vertically aligned graphene electrodes^51^ (a much more conductive aqueous electrolyte was used in the latter case, which unfortunately keeps energy content low). These comparisons highlight the excellent charge transport properties of GN3, which are also reflected in its electrochemical impedance spectroscopic features before and after cycling (Fig. 6b). Based on the modified Frumkin–Melik–Gaykazyan circuit (Fig. S17, ESI†), the intersection of the Nyquist plot with the real axis at the start of the high frequency region corresponded to an equivalent series resistance (*R*_s_) of 3.9 Ω before cycling, which was only marginally increased to 4.4 Ω after 10 000 cycles.

Furthermore, the total absence of semicircles in the high frequency region indicated a very low charge transfer resistivity (*R*_ct_) in the bulk material and at the interfaces.^19,52^ The almost vertical slope of the spectrum in the low frequency region (on the right of the *x*-axis) suggested a highly capacitive behaviour and effective ionic charge transport in the bulk of the electrode material.

**Fig. 7:**
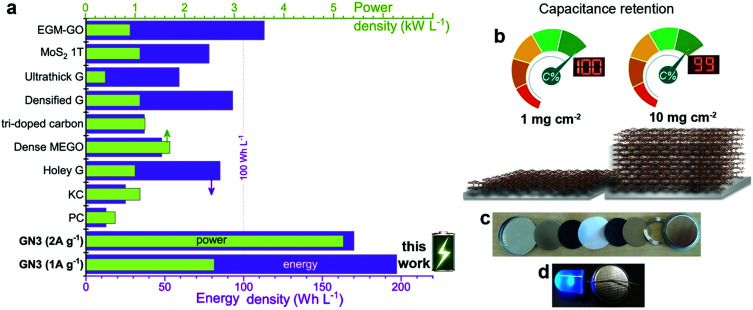
(a) The energy and power density output achieved with GN3 electrodes compared to electrodes made with commercial 2000 m^2^ g^−1^ active carbon and prominent analogues selected from the literature, chosen for their promising features; (holey G: holey graphene;^19^ dense MEGO: compressed, microwave expanded and activated reduced graphene oxide;^17^ tri-doped carbon;^20^ densified G: capillary densified graphene;^18^ ultrathick graphene;^14^ 1T-MoS_2_ (ref. 22) and EGM-GO: exfoliated graphene-mediated graphene oxide^23^). (b) Specific capacitance in symmetric full cell at high (10 mg cm^−2^) mass loading of GN3 was 99% of the recorded capacitance of the low-mass-loading (1 mg cm^−2^) symmetric full cell. (c),(d) Coin cell GN3 electrodes on aluminium foils before (c) and after assembly (d); the assembled cell was used to operate a 4 V LED diode.

Volumetric performance is particularly important for devices in the modern portable energy storage landscape;^2,4,13,14,20,23^ both energy and power density are desired. The former directly affects the amount of energy that can be stored, while high power density enables fast charging and discharging. Energy density is the Achilles heel of supercapacitors, whereas high power densities are one of their greatest strengths, which must be preserved. The GN3 cell (Fig. 5c and Fig. S18, ESI†) demonstrated ground-breaking performance by delivering both ultrahigh energy density and power density. Specifically, its energy density was *ca.* 200 W h L^−1^ at a power of 2.6 kW L^−1^, 170 W h L^−1^ at 5.2 kW L^−1^, and 143 W h L^−1^ at 52 kW L^−1^. To set these results into the context of the current state of the art, they are presented alongside literature data on top-performing materials in Fig. 7a. When making these comparisons, care was taken to ensure that the same set of equations and metrics were used in all cases.^53–55^ The equations from ref. 23 were used, as described in the experimental section. The comparisons highlight the transformative performance of GN3: not only does it have a higher energy density than the previously best-in-class EGM-GO electrode (170 W h L^−1^ for GN3 *vs.* 113 W h L^−1^ for EGM-GO^23^), but this energy could be delivered at a power of 5.2 kW L^−1^ compared to 0.9 kW L^−1^. Importantly, the GN3 cell could also be operated at mass loadings of up to 10 mg cm^−2^, demonstrating almost identical capacitance (Fig. 7b) at a temperature of 38 °C, which is in the range typically used to evaluate energy storage devices,^56–59^ and 81% retention at the same testing conditions as the low mass-loading supercapacitor cell (see experimental part, ESI†). The coin cell shown in Fig. 7c and d, was constructed with a commercial ultrathin 25 μm membrane operating a 4 V LED lamp. Successful operation of carbon materials with such highly attractive features lays the ground for the fabrication of competitive, commercially relevant cells.

## 3. Conclusions

We have discovered a new class of carbon-based materials comprising nitrogen doped graphene with diamond-like tetrahedral bonds for high energy density supercapacitor electrodes that are significantly more dense than comparable materials prepared by mechanical compression,^17^ capillary densification,^18^ and other methods.^19,23^ The new materials are prepared by leveraging the radical-based chemistry of fluorographene, which enables the fruitful combination of sp^2^ and sp^3^ carbon bonds in the same network, along with very high nitrogen doping and vacancies. This hybrid carbon achieves mass densities of 2.8 g cm^−3^, while retaining efficient charge transport, ion penetration, diffusion, and storage. Therefore, cells with electrodes made from these materials offer ground-breaking energy storage capability at very high charging/discharging rates. The discovery of this class of materials will spur intense research on other high-density conductive carbon materials with different functionalities, with the aim of further increasing the competitiveness of supercapacitors in the portable energy storage landscape.

## Conflicts of interest

A European patent with the number EP 3907184 has been published.

## Supplementary Material

EE-015-D1EE02234B-s001
