# Supplementary material for: Nitrogen doped graphene with diamond-like bonds achieves unprecedented energy density at high power in a symmetric sustainable supercapacitor
Source: Energy Environ Sci. 2022 Jan 7;15(2):740–8. doi: 10.1039/d1ee02234b (PMC8848332; doi:10.1039/d1ee02234b)
Supplement: EE-015-D1EE02234B-s001 [file EE-015-D1EE02234B-s001.pdf]

Electronic Supplementary Material (ESI) for Energy & Environmental Science.
This journal is © The Royal Society of Chemistry 2021

# Electronic Supplementary Information

## Nitrogen doped graphene with diamond-like interlayer bonds achieves unprecedented energy density at high power in a symmetric sustainable supercapacitor

Veronika Šedajová,[a,b] Aristides Bakandritsos,*[a,d] Piotr Błoński,[a] Miroslav Medveď,[a] Rostislav Langer,[a,b] Dagmar Zaoralová,[a,b] Juri Ugolotti,[a] Jana Dzíbelová,[a,c] Petr Jakubec,[a] Vojtěch Kupka[a] and Michal Otyepka*[a,e]

[a]Regional Centre of Advanced Technologies and Materials, Czech Advanced Technology and Research Institute (CATRIN), Palacký University, Šlechtitelů 27, 783 71, Olomouc, Czech Republic. Email: michal.otyepka@upol.cz; a.bakandritsos@upol.cz

[b]Department of Physical Chemistry, Faculty of Science, Palacký University, 17. listopadu 1192/12, 779 00 Olomouc, Czech Republic. E-mail: michal.otyepka@upol.cz; a.bakandritsos@upol.cz

[c]Department of Experimental Physics, Faculty of Science, Palacký University Olomouc, 17. listopadu 1192/12, Olomouc, 77900 Czech Republic

[d]Nanotechnology Centre, Centre of Energy and Environmental Technologies, VŠB–Technical University of Ostrava, 17. listopadu 2172/15, Poruba, 708 00 Ostrava, Czech Republic

[e]IT4Innovations, VŠB–Technical University of Ostrava, 17. listopadu 2172/15, 708 00 Ostrava-Poruba, Czech Republic

### Methods

### Reagents and Materials

Graphite fluoride (>61 wt% F), $NaN_3$ (BioXtra) and *N,N*-Dimethylformamide (DMF, ≥98%) were purchased from Sigma-Aldrich. Acetone (pure) and ethanol (absolute) were purchased from Penta, Czech Republic. All chemicals were used without further purification. Ultrapure water was used for preparation of all aqueous solutions. Membra-cell® MD44 dialysis membranes were used for dialysis. Activated carbon from coconut shells (Kuraray YP-80F, 2100 $m^2$ $g^{-1}$) was purchased from Kuraray CO, LTD., Japan. Porous carbon (PC, 2000 $m^2$ $g^{-1}$) was obtained from

ACS Material, LLC (CNP00001). Ultrapure water (18 MΩ $cm^{-1}$) was used for washings and dialysis.

**Synthesis**

Graphite fluoride (1 g) was dispersed in 15 ml DMF and stirred with a teflon coated magnetic stirrer at 500 rpm for 3 days under nitrogen atmosphere in a glass flask. Afterwards, the mixture was sonicated for 4 hours (Bandelin Sonorex, DT255H type, frequency 35 kHz, effective power 160 W), and then left for stirring overnight. Next day, 3 g of $NaN_3$ was added and the dispersion mixture was heated at 130 ºC for 72 h with a condenser and stirring at 800 rpm in an oil bath.

After the end of reaction, the mixture was left to cool down and washing was performed with DMF, acetone, ethanol, hot water (80 $^{o}$C), water, and acidified water (3% solution of HCl). The product was separated from the solvents using centrifugation (Sigma 4-16K) at 13 000 rcf. Finally, washing with water was performed until the material stopped precipitating with centrifuge. Then, the dispersion was subjected to dialysis against ultrapure water, until the conductivity of the dispersion was bellow 0.2 mS $cm^{-1}$.

**Characterization techniques**

Infra-red spectra were measured on an iS5 FTIR spectrometer (Thermo Nicolet), using the Smart Orbit ATR accessory with ZnSe crystal. A drop of a dispersion of the sample in ethanol or water was placed on a ZnSe crystal and left to dry and form a film at ambient environment. Spectra were recorded by summing 50 scans. Nitrogen gas was flowing through the ATR accessory during the background and sample measurements. ATR and baseline correction were used for processing the collected spectra.

X-ray photoelectron spectroscopy (XPS) was carried out with a PHI VersaProbe II (Physical Electronics) spectrometer using an Al $K_\alpha$ source (15 kV, 50 W, spot size 100 µm). All binding energies were referenced to the C1s core level of the C-C bond set to the nominal value of 284.8 eV. The obtained data were evaluated and deconvoluted with the MultiPak (Ulvac-PHI, Inc.) software package. Spectral analysis included a Shirley back-ground subtraction and peak deconvolution employing mixed Gaussian-Lorentzian functions.

Images from transmission electron microscopy were obtained with a JEOL 2100 TEM, equipped with an emission gun of $LaB_6$ type, operating at 160 kV. The high-resolution TEM images were obtained using an FEI Titan electron microscope operating at 80 kV. The samples were also analysed with scanning electron microscopy using Hitachi SU6600 instrument with accelerating voltage of 5 kV. For these analyses, a small droplet of a material dispersion in ultrapure water (concentration approximately 0.1 mg $ml^{-1}$) was placed on a carbon-coated copper grid and left for drying.

The 1H→$^{13}$C and $^{19}$F→$^{13}$C CPMAS NMR measurements were performed using a JEOL spectrometer JNM-ECZ400R with superconducting coil having a magnetic field of 9.4 T (working frequency: 399.8 MHz for $^{1}$H, 376.2 MHz for $^{19}$F and 100.5 MHz for $^{13}$C) equipped with a 3.2 mm MAS probe. The spectra were collected at ambient temperature at spinning rate 18 kHz, using contact time 6 ms for all measurements and relaxation delay 6 s for samples GN3 and GN3-4h, and 5 s for GF, while the number of scans was 13000 for sample GN3, 16000 for sample GN3-4h and 88 for pristine GF.

Raman spectra were obtained on a DXR Raman microscope, the 633 nm excitation line diode laser was used.

Thermal analyses were performed in open $\alpha$-$Al_2O_3$ crucibles with a Netzsch STA 449C Jupiter instrument with an adapted quadrupole mass spectrometer (QMS 403C Aëolos) at a heating rate of 10 °C $min^{-1}$, under an argon or synthetic air flow (100 ml $min^{-1}$) in the sample compartment.

The concentration of sodium was determined using electrothermal atomization atomic absorption spectroscopy ETA-AAS technique, equipped with a graphite furnace (ContrAA 600; Analytik Jena AG, Germany), with a high-resolution Echelle double monochromator (spectral band width, 2 pm at 200 nm). A continuum radiation source was provided by xenon lamp.

XRD measurements were performed using X'Pert PRO MPD diffractometer (PANalytical) in the Bragg–Brentano geometry equipped with a Co X-ray tube (iron filtered Co Kα radiation with $\lambda$=0.178901 nm), fast X'Celerator detector, and programmable divergence and diffracted beam antiscatter slits. Samples were placed on a zero-background Si slide, gently pressed and scanned with a step size of 0.0334°, and the 2θ range of 5°–120° (2θ resolution of 0.017°) was used to record the pattern. Commercially available standards SRM640 (Si) and SRM660 ($LaB_6$)

from NIST were used for line positions and instrumental line broadening evaluation, respectively. The crystalline phases identification was performed employing the High Score Plus (PANalytical) software in conjunction with the PDF-4+ and ICSD databases.

The lateral dimensions of the graphene sheets (La: the in-plane correlation length) and the stack size (Lc) were evaluated using the Scherrer formula (Equation 1).

$$L_{a/c} = \frac{K\lambda}{\beta\cos\theta},$$

where λ is the X-ray wavelength (0.179 nm for CoKa irradiation), θ is the diffraction angle in radians, β is the full width at half maximum (FWHM) of the deconvoluted peak in radians, and K is a constant (K = 1.84 for La (from the 100 reflection) and K = 0.89 for Lc ( from 002 reflection).[1–3]

Raman spectroscopy. The lateral size of the $sp^2$ areas (or the in-plane correlation length) was calculated using the Tuinstra-Koenig equation (ref: [4]):

$$La= C(\lambda)\frac{I_G}{I_D},$$

where C(λ) is a wavelength-dependent constant equal to:

$C(\lambda) = C0+\lambda C1$ ,

where C0 and C1 are also constants, estimated to be −12.6 nm and C1= 0.033, respectively (ref:[5])."

Surface area and pore size analysis was performed by means of $N_2$ adsorption/desorption measurements at 77 K on a volumetric gas adsorption analyser (3Flex, Micromeritics, USA) up to 0.965 $P/P_0$ and for an equilibration time of 40 s. Prior the analysis, the samples were degassed under high vacuum ($10^{-4}$ Pa) at 130 °C for 12 hours, while high purity (99.999 %) $N_2$, and He gases were used for the measurements. The Brunauer–Emmett–Teller area (BET) was determined with respect to Rouquerol criteria for $N_2$ isotherm. Pore size distribution was analysed by N2-NLDFT 77carbon slit pore kernel.

The carbon coated aluminium foil was used as substrate, active material was deposited on it using Dr. Blade technique, dried under infrared-light lamp (Helago® company) and pressed with force of 80 kN between metallic plates for 1 minute. An 18 mm diameter electrode was cut to fit the four-point probe prior to the measurement.

**Electrochemical measurements**

Cyclic voltammetry (CV) and galvanostatic charge-discharge (GCD) were performed on a Bio-Logic instrument (BCS-810) controlled with the BT-Lab software (version 1.64). Electrochemical impedance spectroscopy (EIS) was performed on Metrohm Autolab PGSTAT128N instrument (Metrohm Autolab B.V., Netherlands), controlled with the NOVA software package (version 1.11.2). EIS were recorded using 10 mV amplitude, in the frequency range from 0.1 Hz to 10 kHz at an open circuit potential (OCP). The fitting of recorded curves was performed with the NOVA software package (version 1.11.2), using modified Frumkin-Melik-Gaykazyan circuit with set of resistors (R), capacitors (C), constant phase elements (CPE), Warburg element (W) and coil (because of parasitic inductance) in series and in parallel, as shown in Fig. S16.

For the two-electrode system, symmetrical full-cell supercapacitor device was assembled to evaluate the performance, rate stability and cyclic stability of the product. Note, that the electrodes for the CVs did not have the same mass loading as the electrodes for the GCD tests, because during electrode pasting it is not always possible to get the exact same deposited mass. Briefly, active material was homogeneously dispersed in *N*-methyl-2-pyrrolidone (p.a. ≥ 99%, Sigma-Aldrich) with binder PTFE (Sigma-Aldrich) and conductive carbon (TimCal from MTI) at a ratio of 85:10:5, the mixture was sonicated for 1 hour and mixed using a planetary mixer (Thinky mixer ARV-310, Thinky Corporation) to form homogenous paste. The slurry was pasted on the carbon-coated aluminium foil (Cambridge Energy Solutions, thickness 15 µm) with Dr.'s blade technique (Erichsen, Quadruple Film Applicator, Model 360). Next, the film was dried at 120 °C in vacuum oven overnight, before two electrodes with diameter 18 mm were cut and pressed in between two stainless steel metal plates with force 80 kN for 1 minute (Trystom spol. s.r.o., Olomouc). Afterwards, the mass and thickness of the electrodes was measured with a commercial micrometer (Kinex micrometer, from M & B Calibr, spol. s r.o., Ivančice) and dried again at 120 °C in glass flask with a stopcock under vacuum (40 mbar) for 6 hours. The electrodes in the flask were transferred (under vacuum) to glovebox (Jacomex,

argon atmosphere) to avoid any humidity. For assembly of the supercapacitor device and testing, the two electrodes (loaded material 1.4 and 1.4 mg, respectively) and same thickness (2 µm each) were placed in a sleeve (El-Cell insulator sleeves equipped with Whatman® glass microfiber paper separator with thickness 0.26 mm). The separator membrane was soaked with 90-100 µl of electrolyte. A mixture of 1-ethyl-3-methylimidazolium tetrafluoroborate (EMIM-$BF_4$, from Sigma Aldrich, ≥99.0% (HPLC)) and 1,1,2,2-tetrafluoroethyl-2,2,3,3-tetrafluoropropyl ether (TTE from Tokyo Chemical Industry, >95.0%) in ratio 9:1 was used as electrolyte, which were dried for 2 days prior use with molecular sieves. The electrodes were enclosed and pressed inside the sleeve with stainless steel plungers, and whole device was tightened and connected (El-Cell PAT-cell for organic electrolytes) into the docking station. For the cycling stability test, more intensive drying was employed; the electrodes were dried at 130 °C in a glass flask with a stopcock under vacuum (10 mbar) for 8 hours and the electrolyte was dried for 1 week with molecular sieves prior use.

Before actual testing of material, conditioning was performed as follows:

Hold of potential for 5 minutes at 1.2 V, 10 cycles at current density 0.5 A $g^{-1}$ up to 2 V, 10 cycles at current density 1 A $g^{-1}$ up to 3.7 V, 5 cycles of current densities 3 A $g^{-1}$, 5 A $g^{-1}$ and 7 A $g^{-1}$.

Specific capacitance of the active material (amount of GN3 material with binder and conductive additive deposited on the electrodes) ($C_s$, F·$g^{-1}$) was calculated from GCD discharge curve according to previous reports[6]:

$$C_s = 2 \times \frac{I \cdot t}{m \cdot V} \text{ [F/g]}; \qquad C_v = C_s \cdot \frac{m}{V_{el}} \text{ [F cm}^{-3}\text{]}$$

Gravimetric and volumetric energy density and power density were calculated as follows:

$$E_g = \frac{1}{2 \times 4} \cdot \frac{C_s \cdot V^2}{3.6} \text{ [Wh kg}^{-1}\text{]}; \qquad P_g = \frac{E_g}{t} \cdot 3600 \text{ [W kg}^{-1}\text{]}; \qquad E_v = \frac{1}{2 \times 4} \cdot \frac{C_v \cdot V^2}{3.6} \text{ [Wh L}^{-1}\text{]};$$

$$P_v = \frac{E_v}{t} \cdot 3600 \text{ [W L}^{-1}\text{]}$$

where $I$ is the discharge current, $t$ is discharging time (IR-drop was excluded), $m$ is the mass of the material on one electrode (including binder and conductive additive), $V$ is potential window, $V_{el}$ is the volume of electrode material on one electrode.

For the high mass loading tests (10 mg cm$^{-2}$), a similar procedure was used to prepare the paste, with the increased ratio of conductive additive in the slurry (final ratio was 80:10:10, corresponding to GN3 material, PTFE and TimCal, respectively). The mass of loaded material on electrodes was 25 mg and 25.1 mg, respectively. The separator was wetted with a mixture of 1 M solution of tetraethylammonium tetrafluoroborate (99%, Sigma Aldrich) in acetonitrile (anhydrous, 99.8%, Sigma Aldrich) and EMIM-$BF_4$ in ratio of 50:50. The testing was performed at 38 °C.[7] To verify the high mass density of the GN3 material at the high-mass loading as well, 4.2 mg of GN3 was deposited on a carbon coated aluminium foil of a diameter of 0.8 cm (mass = 1.7 mg) and placed in a common die for preparing pellets (Pike Technologies). The pressing was performed with a force of 100 kN using Shimadzu AGS-X 100 kN. After pressing, the mass and the thickness of the electrode were measured, and a mass density of 2.7 g cm$^{-3}$ was calculated for the deposited material. The final electrode mass loading was 8.3 mg cm$^{-2}$.

Comparative testing was also performed at 60 °C using the same conditions as for the low mass loading.

All energy and power densities mentioned in the manuscript from materials reported in the literature comply to the equations reported above. All numbers reported were verified independently from the discharge times given in every published article.

**Computational details**

To explore possible reaction pathways in the initial phase of the N-doping of fluorographene with $NaN_3$ in DMF (Fig. S3, ESI), finite-size density functional theory (DFT) calculations with Gaussian09 program[8] were performed. Ground state structures of all investigated species were optimized by the ωB97X-D method[9] in combination with 6-31++G(d,p) basis set. For open-shell systems, the spin-unrestricted formalism has been applied. The solvent effects were simulated using the universal continuum solvation model based on electron density (SMD).[10] To mimic the semilocal flexibility of FG sheets, the FG-like structures were obtained by constrained geometry optimizations keeping the edge carbon atoms frozen. The structures of single and double vacancies (SV and DV) were taken from ref.[11].

The impact of N-doping on the development of C-C $sp^3$ bonds in smaller bi-layered models was studied at the PBE0-D3/6-31++G(d,p) level of theory[12,13], which describes intermolecular interactions consistently with the DFT/plane wave approach employing the periodic-boundary conditions which was used to study larger bi-layered models as described below.

The first-principles spin-polarized DFT calculations were performed by applying the Perdew-Burke-Ernzerhof functional (PBE)[14] with inner electrons represented as projector augmented waves (PAW)[15,16] as implemented in Vienna Ab initio Simulation Package (VASP).[17,18] The wave functions were expanded in plane waves with a cut-off kinetic energy of 400 eV. The Brillouin zone was sampled with a 1 × 1 × 1 Γ-centered Monkhorst–Pack k-point mesh.[19] All the optimized structures were converged to forces of less than $10^{-2}$ eV/Å and energy below $10^{-6}$ eV, respectively, in the self-consistent field (SCF) cycle. Graphene was modelled as $C_{192}H_{48}$ and $C_{640}H_{88}$ flakes. DFT-D3 method of Grimme[13] was utilized to account for the dispersion corrections.

# Supplementary Figures and Tables

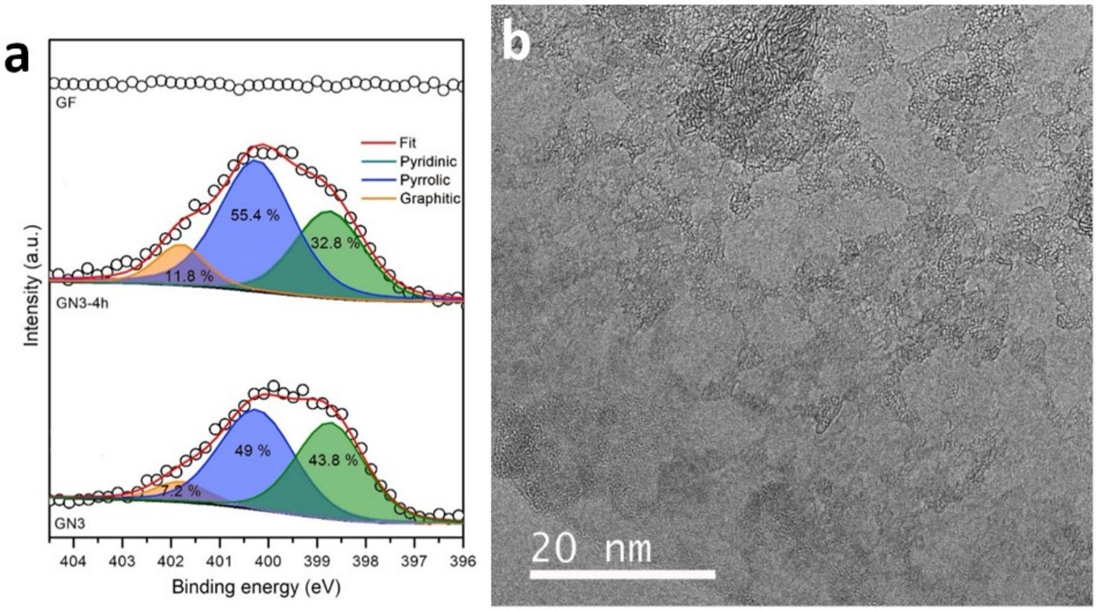


**Fig. S1** (a) HR-XPS deconvoluted spectra for the N1s region of the GN3 derivatives after 4h (GN3-4h) and 72h of reaction (GN3). The increase of the pyridinic type nitrogens is in keeping with the lower energy state and higher stability of the pyridinic configurations.[11] (b) HR-TEM image of GN3 material, showing its holey structure.

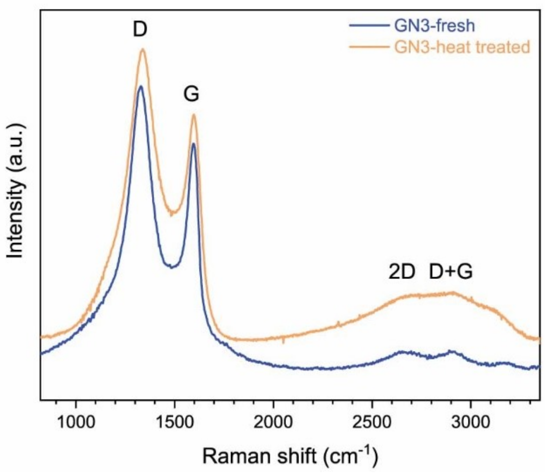


**Fig. S2** Raman spectrum of GN3 (bottom blue line) and heat treated in argon atmosphere at 1000 °C (top orange line).

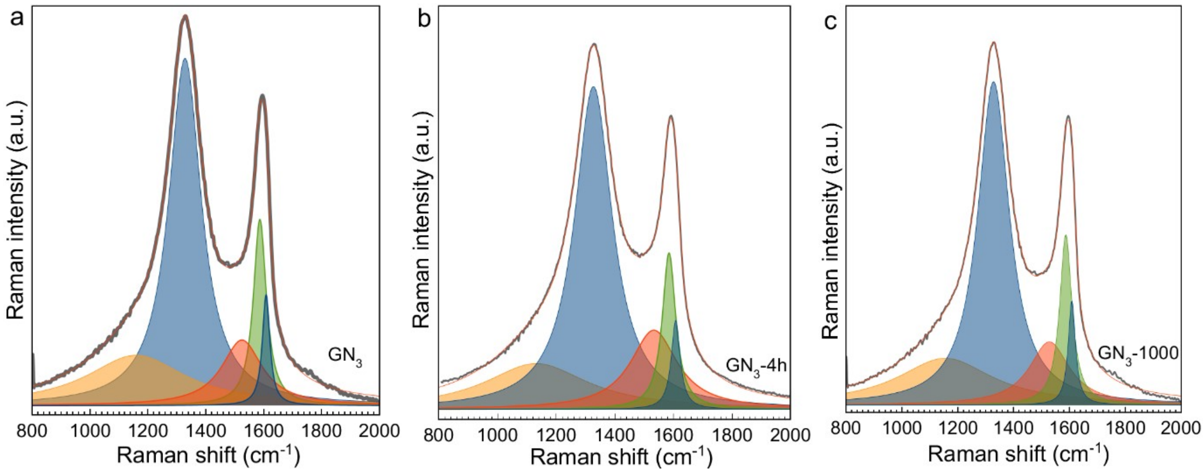


**Fig. S3**. Raman Spectra of the (a) GN3-4h ($I_D/I_G$=1.9, $L_a$=4.4 nm), (b) GN3 ($I_D/I_G$=2.1, $L_a$=4.0 nm) and (c) $I_D/I_G$=1.9, $L_a$=4.3 nm).

**Comments**: Raman spectra of GN3 were analyzed more elaborately, in order to provide further macroscopic information on the structure. Typically, the first order Raman scattering spectra from both amorphous and graphene derivatives[14–17] are successfully interpreted with deconvolution of the D (around 1350 $cm^{-1}$) and G (around 1590 $cm^{-1}$) bands. Both D and G bands are connected to the $sp^2$ rings (D to $sp^2$ rings with adjacent $sp^3$ defects, and G to crystalline/conjugated $sp^2$ islands),[18] and since Raman spectroscopy is several-fold more sensitive to $sp^2$ areas than to $sp^3$ ones, the D and G bands dominate even in amorphous/active carbons with small content in $sp^2$ areas.[18] The GN3 spectrum is also dominated by these bands (Fig. S3) including the 4h GN3 intermediate (GN3-4h), and GN3 after annealing in inert atmosphere at 1000 °C (GN3-1000). The deconvolution of the D and G bands and estimation of lateral size, $L_a$, (see experimental part bellow for calculation details) corroborate the small lateral size (4 nm) of the aromatic areas, as obtained from XRD. Very interestingly, the comparison of GN3 before and after annealing at 1000 °C, revealed the same Raman features (also reflected in the respective $I_D/I_G$ and $L_a$ values in Fig. S3). This indicates that no healing process of the graphene backbone takes place during annealing and that the same amount and type of defects are preserved before and after the annealing. This corroborates the extensive holey structure observed from the TEM characterization, giving rise to permanent defects due to large vacancies and $sp^3$ bonds.

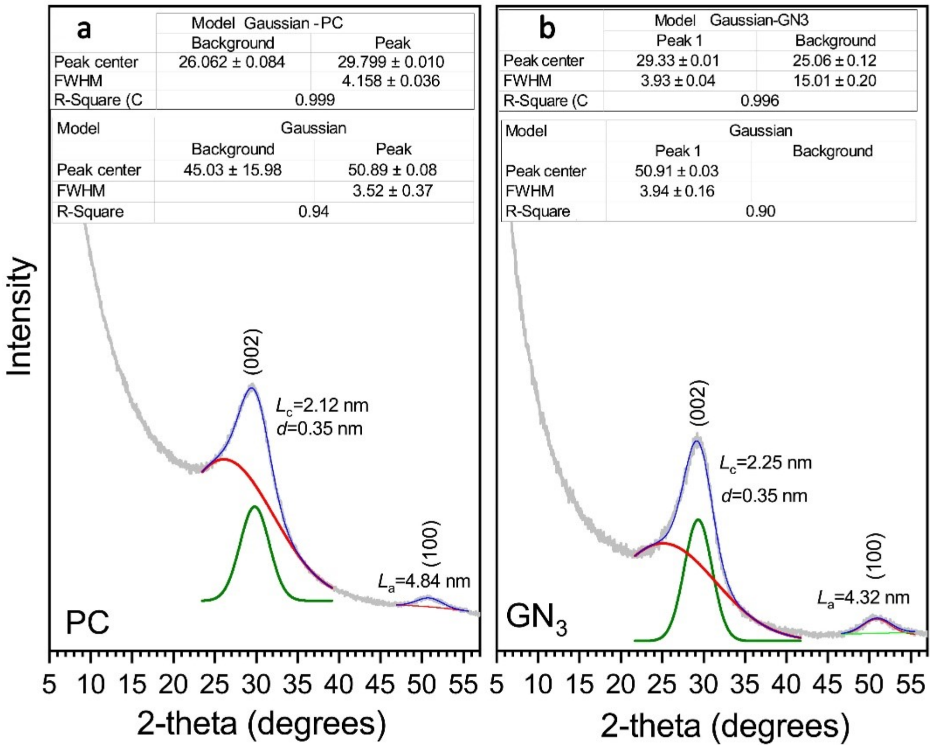


**Fig. S4**. X-ray diffraction patterns of (a) Commercial active porous carbon (PC), and (b) the $GN_3$ product (Co Ka radiation of 1.79 Å).

**Comments**: X-ray diffraction (XRD) was performed on GN3, and on the commercial porous carbon, for comparison (PC, Fig. S4). In both cases, the broad bump at the 30° region of the (002) reflection was fitted with two Gaussian peaks, typical for disordered carbons,[11] originating from the diffraction from aromatic graphenic layers (narrow diffraction), and from amorphous $sp^3$ carbon areas (broader diffraction). From the narrow diffraction and the Scherrer formula, the $L_c$ average stack height was obtained, while from the (100) diffraction the $L_a$ average lateral size of the graphenic platelets was obtained. $L_c$ and $L_a$ values of GN3 (Fig. S4b) were very similar with that of other disordered carbons,[12] pointing to the small extent of ordering and limited planarity of the aromatic areas, in agreement with the high content of $sp^3$ carbons deduced from NMR. Thus, GN3 displays a significantly disordered structure with vacancies (holes) and tetrahedral carbon bonds developed randomly, without showing an interlayer spacing of 0.29 Å. Such disordered XRD patterns have been observed upon the defluorination of fluorinated graphites,[9,13] as well as in carbon fibers, whereby the initial sharp and intense 002 graphitic reflection, after fluorination and defluorination, resulted to very broad, amorphous carbon-like 002 reflection at ca. 0.35 nm (all experimental details and equations used in the analysis of the XRD results are provided later bellow).

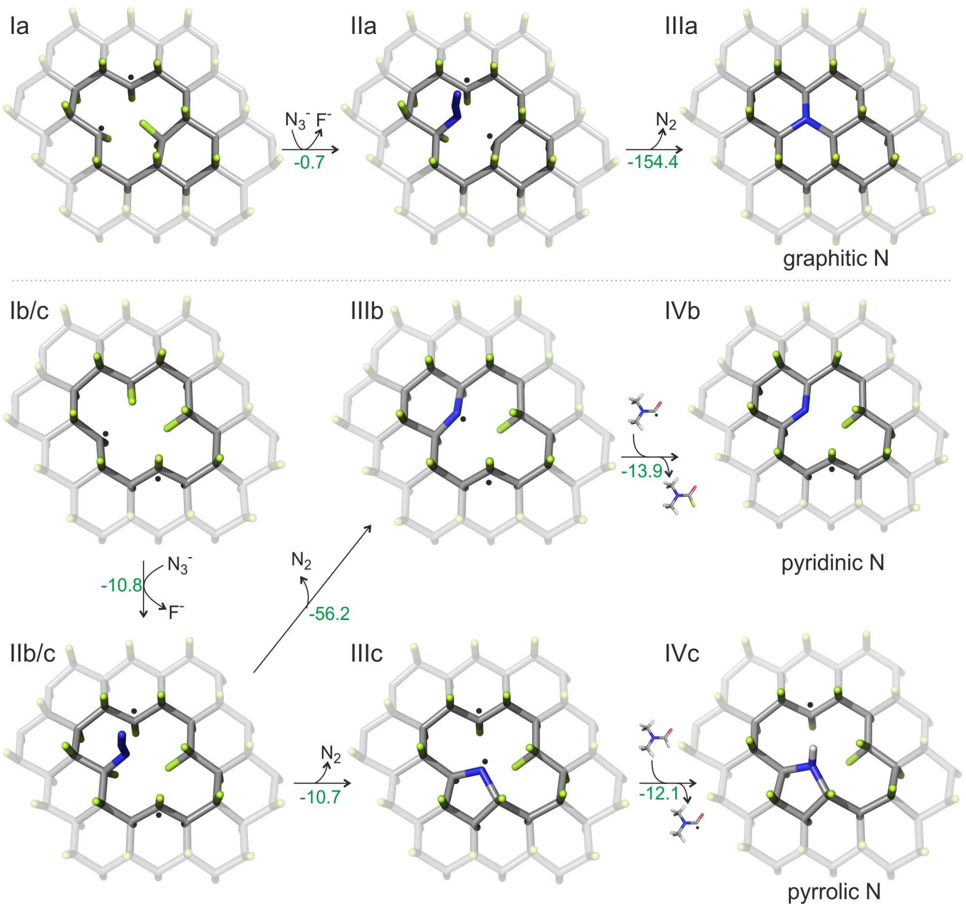


**Fig. S5** Formation of N-doped graphene structures by the reaction of FG with sodium azide in DMF modelled at the ωB97X-D/6-31++G(d,p)/SMD(solvent=DMF) level of theory.[9,10] (a) The formation of graphitic nitrogen configuration by a nucleophilic attack of the azide anion on the single vacancy (SV). (b) The formation of pyridinic and pyrrolic nitrogen configuration by a nucleophilic attack of the azide anion on a double vacancy (DV). The reaction energies are given in kcal mol$^{-1}$. The general reaction mechanism is in line with the recent understandings of the chemistry of fluorographene.[11,20–22]

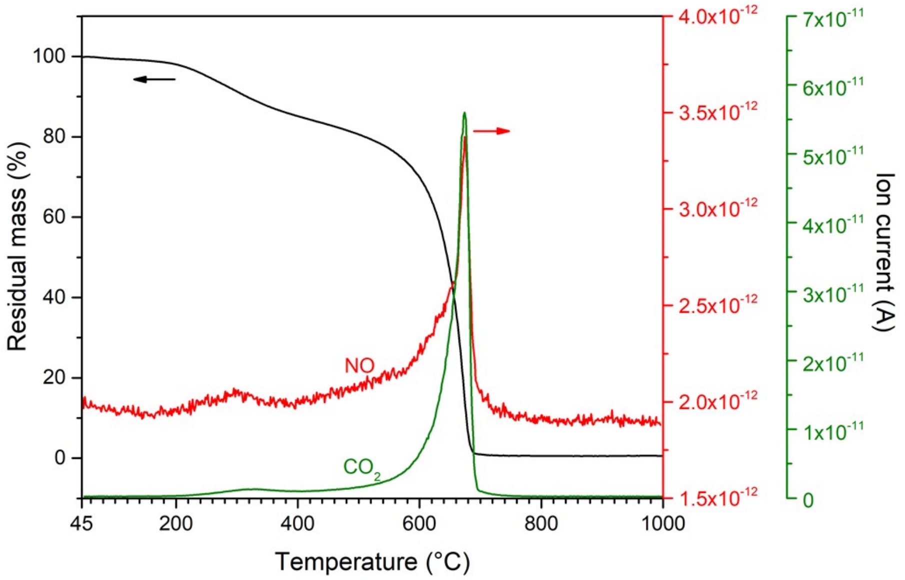


**Fig. S6** Thermogravimetric and evolved gas analysis with mass spectroscopy of GN3 in air with NO and $CO_2$ channels for the monitored mass fragments.

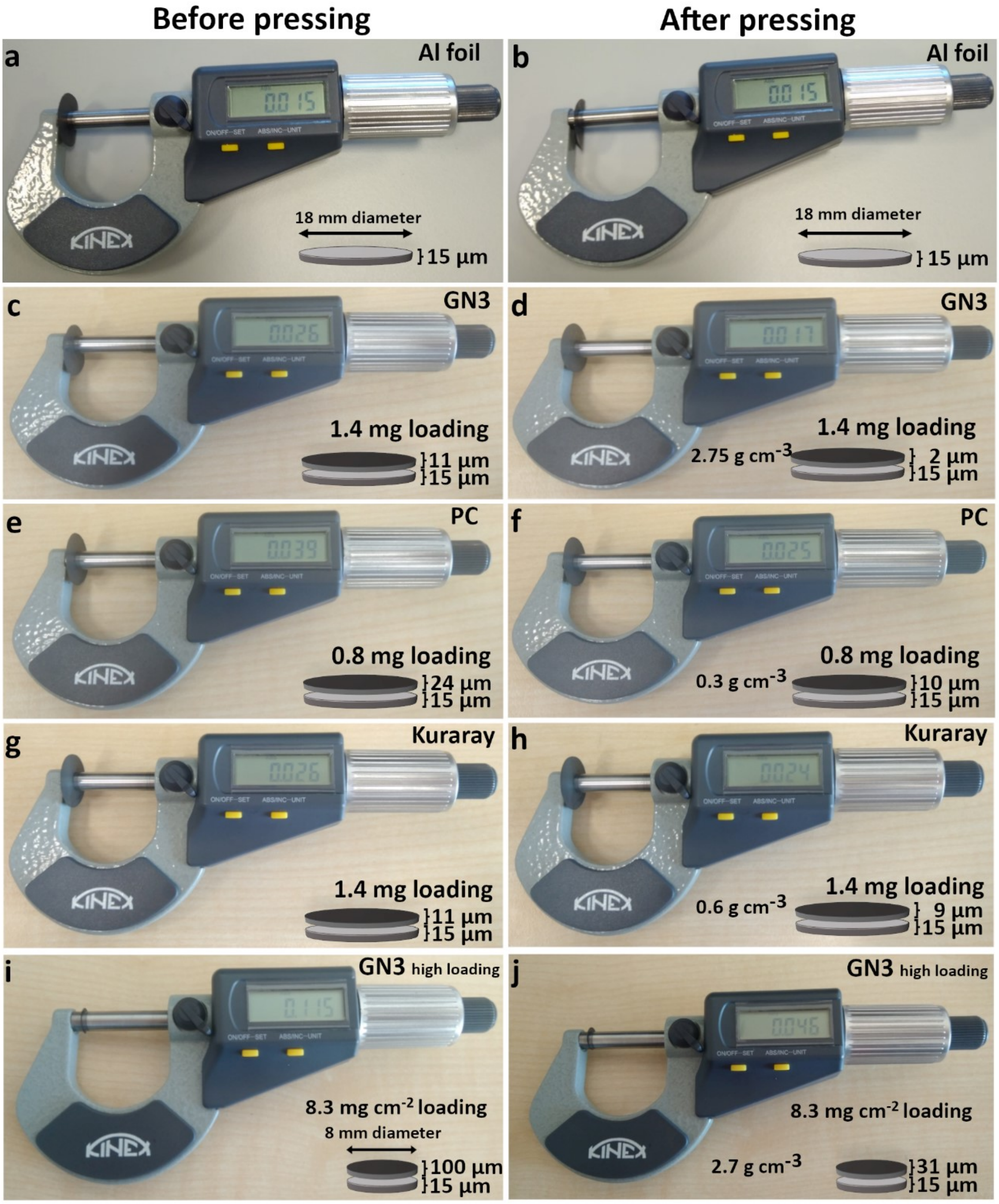


**Fig. S7** Photos of films pasted on Al foils measured with a digital micrometer and showing the thicknesses before and after pressing at 80 kN (panels a-h) and 100 kN (panels i,j) for 1 min. (a),(b) bare Al foil. (c),(d) GN3 film on Al foil. After pressing the density increased from 0.5 g cm$^{-3}$ to 2.75 g cm$^{-3}$. (e),(f) Porous carbon on Al foil. After pressing the density increased from 0.125 g cm$^{-3}$ to 0.3 g cm$^{-3}$. (g),(h) Kuraray carbon on Al foil. After pressing the density increased from 0.5 g cm$^{-3}$ to 0.6 g cm$^{-3}$. (i),(j) High mass GN3 film on Al foil. After pressing the density increased from 0.84 g cm$^{-3}$ to 2.7 g cm$^{-3}$. Smaller electrodes were used in this case because the initial 100 µm film was not stable when placed inside a die for pellet preparation.

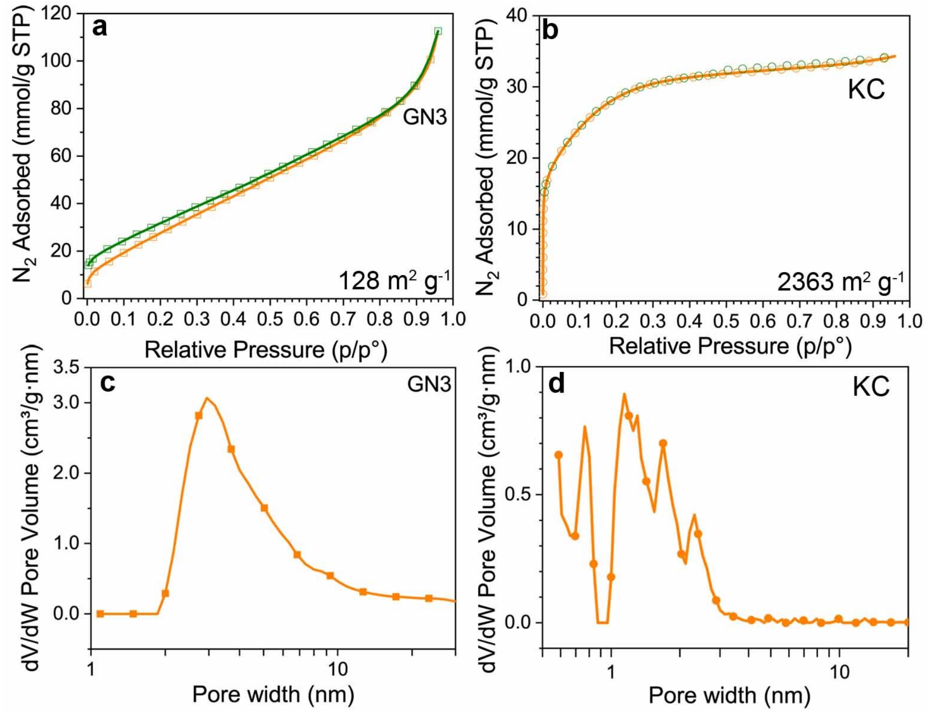


**Fig. S8** Nitrogen adsorption and desorption isotherms of (a) GN3 and (b) commercial high surface-area active carbon used in supercapacitors (KC, Kuraray YP-80F), recorded at 77 K. The measured surface-area of KC is practically identical with the one reported from the provider (2100 $m^2$ g, ref. [23]). The low pressure hysteresis observed for GN3 (despite the 40 s equilibration time allowed during sorption/desorption) constitutes the pore size analysis relatively unsafe and can be related to various reasons, including changes of the pore size or strong sorbent/adsorbate interactions,[24] or it can be probably due to requirement of very long equilibration time during the desorption.[25] The shape of the isotherm with the very small knee at the low pressure regions clearly suggests absence of micropores. (c),(d) Pore width distribution curves comparing GN3 and KC, showing more uniform pore-size for GN3 material, compared KC carbon.

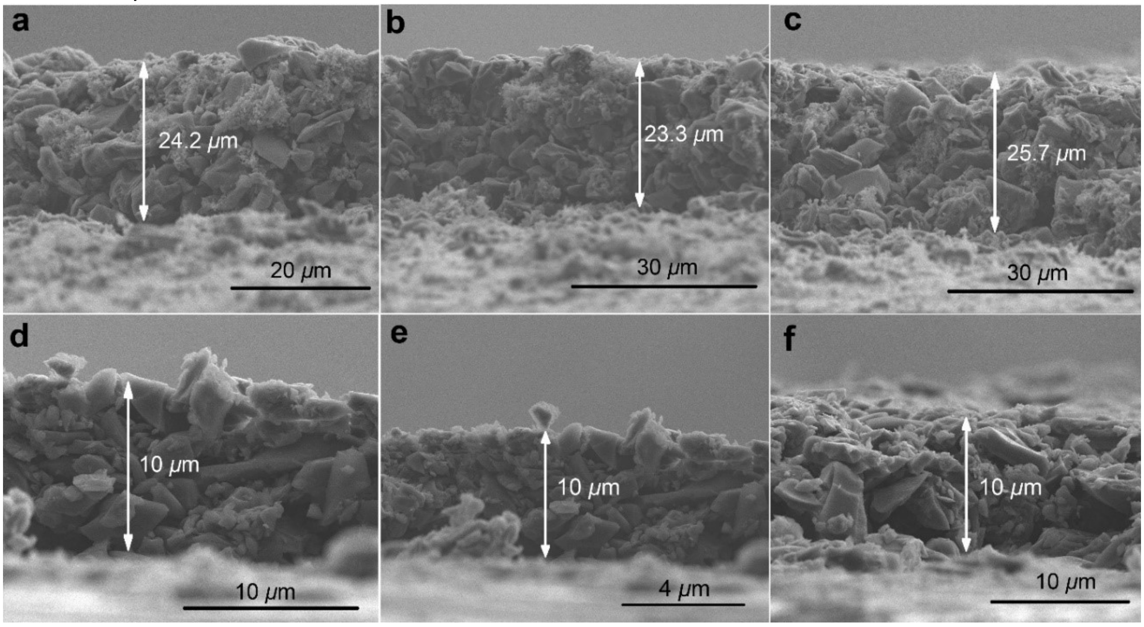


**Fig. S9** SEM images of the high surface area (2000 $m^2$ $g^{-1}$) PC microporous carbon[26] electrode films from ACS materials (a)-(c) before and (d)-(f) after compression. The films were prepared under identical conditions as the GN3-based films.

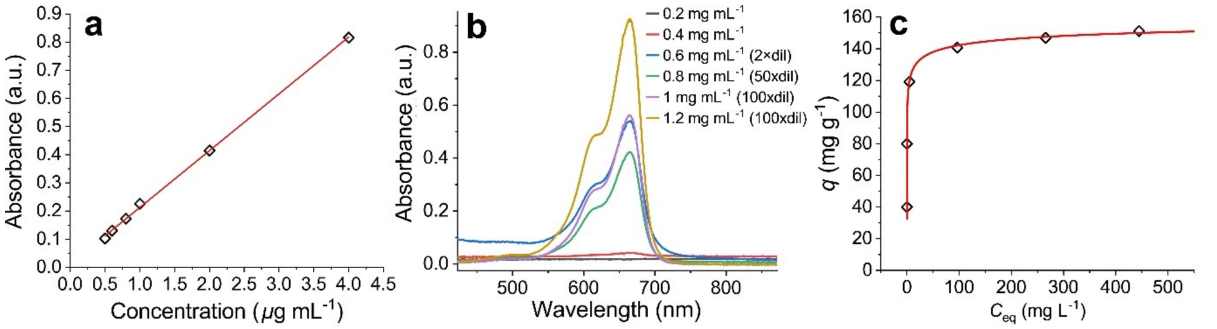


**Fig. S10** Methylene blue adsorption on GN3. (a) Calibration curve obtained from methylene blue standard solutions based on the UV-Vis absorption spectra, (b) UV-Vis absorption spectra of methylene blue which was not adsorbed from GN3 dispersions of 5 mg $mL^{-1}$. The initial amounts of MB are shown on the graph. (c) From the data of panel "b" the adsorbed amount of MB was calculated per g of GN3 ($q$), and the equilibrium concentration of MB ($C_e$). The data were fitted with the extended Langmuir isotherm, $q = \frac{q_{max}Kq_e^{\,c}}{1+Kq_e^{\,c}}$, where $q_{max}$ (mg $g^{-1}$) is the maximum adsorption capacity of GN3, $K$ is the equilibrium constant (L $mg^{-1}$). According to the fitting results the following values were obtained, $q_{max}$=170 mg $g^{-1}$, K=1.6 L $mg^{-1}$, $c$=0.25, and a reduced chi-square of 2.4. The experimental $q_{max}$ was 153 mg $g^{-1}$, according to which the surface area of $GN_3$ was 300 $m^2$ $g^{-1}$ (considering a 319.85 $Å^2$ area for the MB molecule).

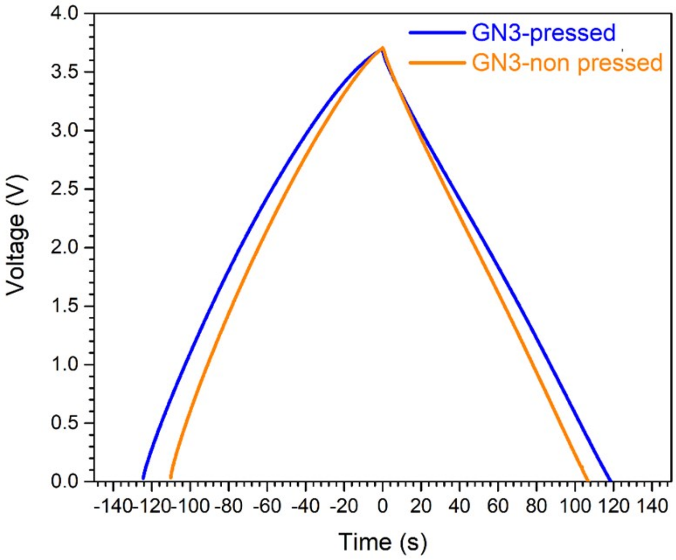


**Fig. S11** GCD for a pressed GN3 electrode (blue line) and non-pressed electrode (orange line). Tests were performed in symmetric full-cells in the EMIM-$BF_4$ and TTE (9:1) electrolyte.

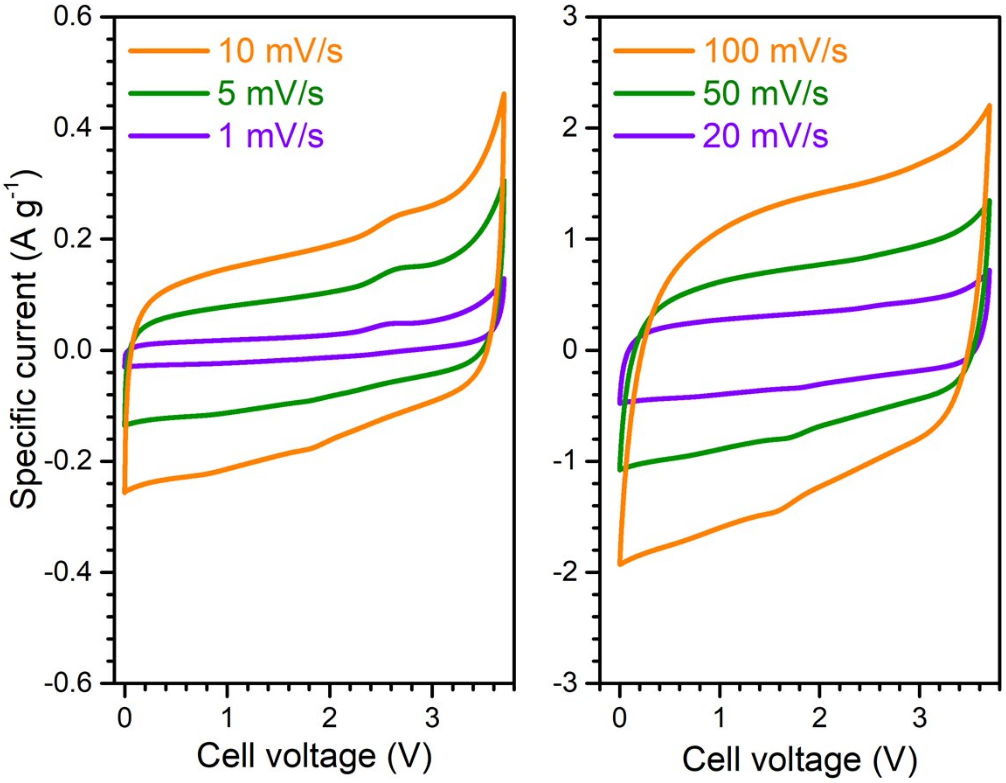


**Fig. S12** CV curves for the GN3 electrode material in the EMIM-$BF_4$ and TTE (9:1) electrolyte at various scan rates, in symmetric full-cell.

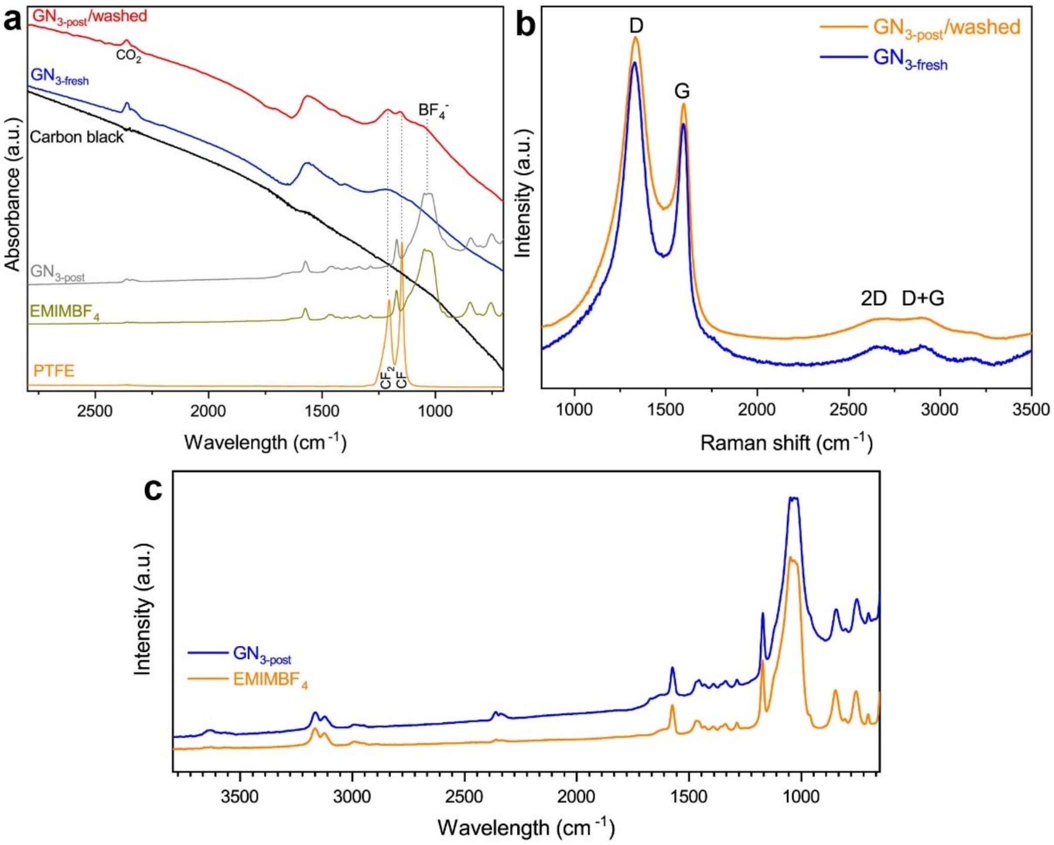


**Fig. S13** (a) Infra-red spectra of the GN3 material after assembly ($GN_{3\text{-fresh}}$) and after cycling ($GN_{3\text{-post/washed}}$). The spectra of the electrolyte (EMIM-$BF_4$) and of the binder (PTFE) which were used are also shown, in order to indicate the bands in the $GN_{3\text{-post/washed}}$ electrode material which do not originate from the material itself. Considering this, it is evident that the spectrum of the GN3 material after cycling is practically identical to the fresh one, verifying its structural stability. (b) Raman spectra of the GN3 electrodes before and after cycling. (c) Infra-red spectra showing the fresh electrolyte and the electrolyte adsorbed on the electrode after cycling 10000 times (spectra obtained without washing the electrode). The two spectra are identical, also verifying the stability of the electrolyte.

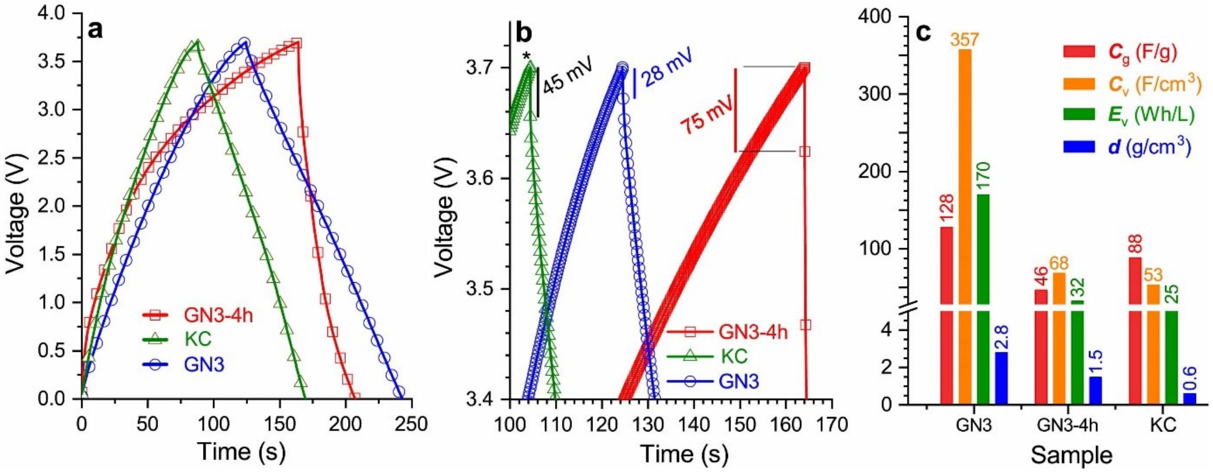


**Fig. S14** (a) GCD curves at 2 A $g^{-1}$, (b) internal resistance drop (focused region of GCD curves of panel "a" on the start of the discharge; the asterisk denotes that the GCD for KC carbon has been shifted to the right of the X-axis for visibility reasons, while the original values appear in panel "a"); (c) Comparison of the performance of the final GN3 (72h) product, with the intermediate nitrogen-doped graphene (GN3-4h) and with the KC carbon.

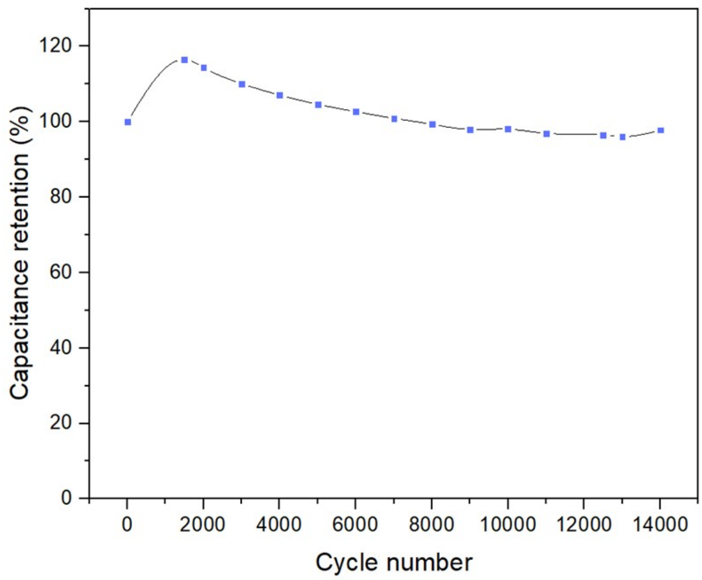


**Fig. S15** Cycling stability of GN3 material at 5 A $g^{-1}$ for 14 000 cycles with 98 % capacitance retention.

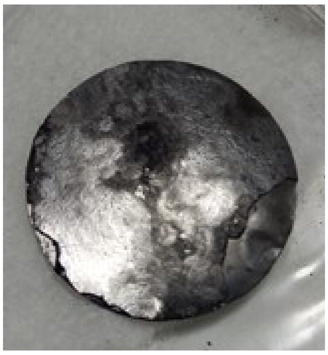

**Fig. S16** A 200 µm GN3 pressed film, showing a shiny surface, indicative of high conductivity.

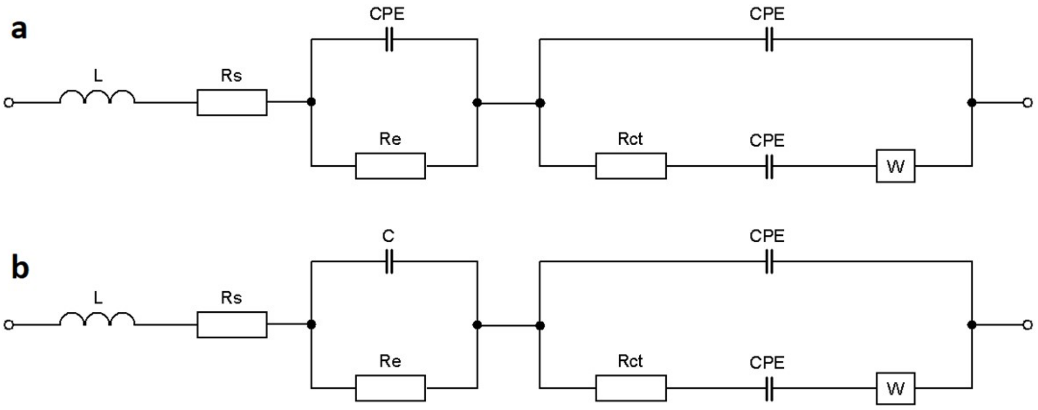


**Fig. S17** Modified Frumkin-Melik-Gaykazyan circuits, used for fitting of EIS curves recorded (a) after assembling of full-cell, (b) after cycling of material at 10 A $g^{-1}$ for 10,000 cycles.

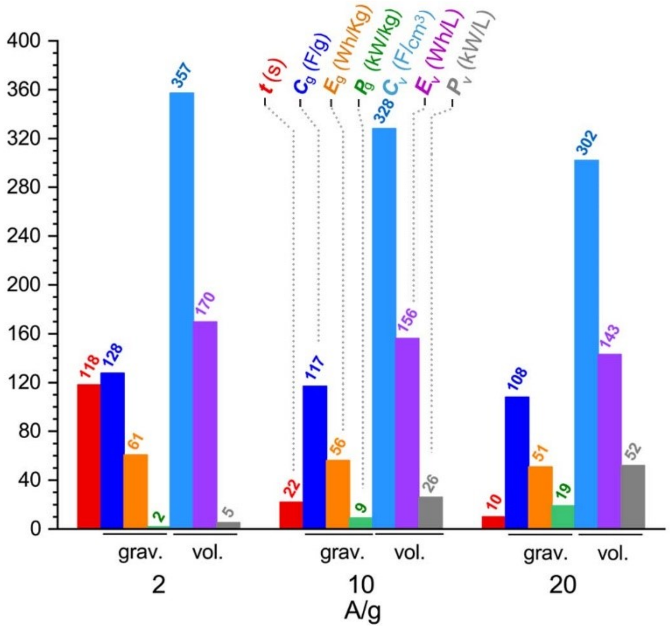


**Fig. S18** Performance characteristics of the symmetric full-cell built from the GN3 material showing the discharge time ($t$), specific capacitance ($C_g$), specific energy and power ($E_g$ and $P_g$ respectively) as well as the volumetric capacitance ($C_v$), energy and power densities ($E_v$ and $P_v$ respectively).

**References.**


1 T. Ariyanto, J. Glaesel, A. Kern, G.-R. Zhang and B. J. M. Etzold, *Beilstein J. Nanotechnol.*, 2019, **10**, 419–427.

2 D. J. Lim, N. A. Marks and M. R. Rowles, *Carbon*, 2020, **162**, 475–480.

3 B. Feng, S. K. Bhatia and J. C. Barry, *Energy Fuels*, 2003, **17**, 744–754.

4 F. Tuinstra and J. L. Koenig, *J. Chem. Phys.*, 1970, **53**, 1126–1130.

5 M. J. Matthews, M. A. Pimenta, G. Dresselhaus, M. S. Dresselhaus and M. Endo, *Phys. Rev. B*, 1999, **59**, R6585–R6588.

6 Z. Li, S. Gadipelli, H. Li, C. A. Howard, D. J. L. Brett, P. R. Shearing, Z. Guo, I. P. Parkin and F. Li, *Nat. Energy*, 2020, **5**, 160–168.

7

8 M. J. Frisch, G. W. Trucks, H. B. Schlegel, G. E. Scuseria, M. A. Robb and J. R. Cheeseman, 2016, Gaussian, Inc., Wallingford CT.

9 J.-D. Chai and M. Head-Gordon, *Phys. Chem. Chem. Phys.*, 2008, **10**, 6615–6620.

10 R. Ditchfield, W. J. Hehre and J. A. Pople, *J. Chem. Phys.*, 1971, **54**, 724–728.

11 D. Zaoralová, V. Hrubý, V. Šedajová, R. Mach, V. Kupka, J. Ugolotti, A. Bakandritsos, M. Medved' and M. Otyepka, *ACS Sustain. Chem. Eng.*, 2020, **8**, 4764–4772.

12 C. Adamo and V. Barone, *J. Chem. Phys.*, 1999, **110**, 6158–6170.

13 S. Grimme, J. Antony, S. Ehrlich and H. Krieg, *J. Chem. Phys.*, 2010, **132**, 154104.

14 J. P. Perdew, K. Burke and M. Ernzerhof, *Phys. Rev. Lett.*, 1996, **77**, 3865–3868.

15 G. Kresse and D. Joubert, *Phys. Rev. B*, 1999, **59**, 1758–1775.

16 P. E. Blöchl, *Phys. Rev. B*, 1994, **50**, 17953–17979.

17 G. Kresse and J. Furthmüller, *Phys. Rev. B*, 1996, **54**, 11169–11186.

18 G. Kresse and J. Furthmüller, *Comput. Mater. Sci.*, 1996, **6**, 15–50.

19 H. J. Monkhorst and J. D. Pack, *Phys. Rev. B*, 1976, **13**, 5188–5192.

20 M. Medveď, G. Zoppellaro, J. Ugolotti, D. Matochová, P. Lazar, T. Pospíšil, A. Bakandritsos, J. Tuček, R. Zbořil and M. Otyepka, *Nanoscale*, 2018, **10**, 4696–4707.

21 W. Lai, D. Xu, X. Wang, Z. Wang, Y. Liu, X. Zhang, Y. Li and X. Liu, *Phys. Chem. Chem. Phys.*, 2017, **19**, 24076–24081.

22 U. Rajeena, P. Raveendran and R. M. Ramakrishnan, *J. Fluor. Chem.*, 2020, **235**, 109555.

23 Shanghaihuipingnewenergyco.,Ltd.. [online], <http://www.scmbattery.com/en/about.php>.

24 K. S. W. Sing, *Pure Appl. Chem.*, 1985, **57**, 603–619.

25 A. M. Silvestre-Albero, J. M. Juárez-Galán, J. Silvestre-Albero and F. Rodríguez-Reinoso, *J. Phys. Chem. C*, 2012, **116**, 16652–16655.

26 Porous Carbon, https://www.acsmaterial.com/porous-carbon.html, (accessed May 30, 2020).
